# Micromorphological details and identification of chitinous wall structures in *Rapana venosa* (Gastropoda, Mollusca) egg capsules

**DOI:** 10.1038/s41598-020-71348-5

**Published:** 2020-09-03

**Authors:** Verginica Schröder, Ileana Rău, Nicolae Dobrin, Constanţa Stefanov, Ciprian-Valentin Mihali, Carla-Cezarina Pădureţu, Manuela Rossemary Apetroaei

**Affiliations:** 1 Department of Cellular and Molecular Biology, Faculty of Pharmacy, “Ovidius” University, Capitan Aviator Al. Serbanescu Street No. 6, Campus C, Constanta, Romania; 2 Department of General Chemistry, Faculty of Applied Chemistry and Materials Science, University Politehnica of Bucharest, Polizu Street No. 1, Bucharest, Romania; 3 Electron Microscopy Department and Center for Research and Development of the Morphological and Genetic Studies of Malignant Pathology, “Ovidius” University, University Street No. 1, Campus B, Constanta, Romania; 4 Department of Life Sciences, Faculty of Medicine, “Vasile Goldiș” Western University From Arad, Arad, Romania; 5 Department of Naval and Port Engineering and Management, “Mircea Cel Batran” Naval Academy, Fulgerului Street No. 1, Constanta, Romania

**Keywords:** Biopolymers in vivo, Membrane structure and assembly, Electron microscopy

## Abstract

The present study evaluated the structural and ultrastructural characteristics of *Rapana venosa* egg capsules, starting from observations of their antifouling activity and mechanical resistance to water currents in mid-shore habitats. Optical microscopy, epifluorescence, and electron microscopy were used to evaluate the surface and structure of the *R. venosa* egg capsules. These measurements revealed an internal multilamellar structure of the capsule wall with in-plane distributions of layers with various orientations. It was found that the walls contained vacuolar structures in the median layer, which provided the particular characteristics. Mechanical, viscoelastic and swelling measurements were also carried out. This study revealed the presence and distribution of chitosan in the capsule of *R. venosa*. Chitosan identification in the egg capsule wall structure was carried out through SEM–EDX measurements, colorimetric assays, FT-IR spectra and physical–chemical tests. The biopolymer presence in the capsule walls may explain the properties of their surfaces as well as the mechanical resistance of the capsule and its resistance to chemical variations in the living environment.

## Introduction

*Rapana venosa* (Valenciennes, 1846) is an invasive marine neogastropod in Europe and North and South America. Native to the Sea of Japan, Yellow Sea, East China Sea and Bohai Bay, *R. venosa* was accidentally introduced through ballast water into the Mediterranean Sea, Azov Sea, Adriatic Sea, Aegean Sea and Black Sea^1,2^. In particular, these gastropods reached the Black Sea in the 1940s through egg capsules attached to ship hulls. The accommodation and growth of the species in the Black Sea was facilitated by the local environmental conditions, which favoured their reproduction and development. *R. venosa* is a predator that feeds on oysters and mussels in shallow zones of 40–60 m in depth. The transatlantic expansion of *R. venosa* in Chesapeake Bay on the Mid-Atlantic coast of the United States^3^ and Rio de la Plata between Uruguay and Argentina prompted biological and ecological studies to understand its spread^4^.

*Rapana venosa* eggs are released at various depths and are protected by capsules. The egg capsules are arranged in clusters and attached to various substrates (rocks, shells, algae and ships). These egg capsules are 12–18 mm long and are arranged in a single deposit. Each tube may include thousands of eggs^5^, whose development is controlled by environmental factors (temperature, oxygen level and salinity) and by the resistance of the capsule. The capsule structure is adapted to offer mechanical protection against intertidal and subtidal variations. Embryonic capsules provide protection against bacteria and UV light^6^ and influence gas exchange^7^. In addition, these capsules provide resistance to temperature and salinity variations^3^ and protection against different predators^8^.

Studies on the structure of these capsules in mollusc species have revealed a complex composition of proteins and polysaccharides with variable consistency in *Dicathais orbita*^9^, *Ilyanassa obsoleta*^10^, *Chicoreus virgineus* and *Hemifusus pugilinus*^11^. In marine gastropods, benthic egg capsules have been documented for the whelk families Muricidae, Buccinidae and Nassariidae^12^. Animal egg encapsulation has evolved independently in several distinct phylogenetic groups in response to risks faced by embryonic development and has been shown to have a major evolutionary impact on the reproductive success and habitat expansion of many taxonomic groups^8^.

Independent evolution also results in adaptive solutions being often dissimilar among various phylogroups. For this reason, knowledge concerning the mechanisms of adaptation in one phylogroup cannot be simply extrapolated to other groups. This explains our interest in the particular adaptations of the egg capsule structure and composition in *R. venosa*.

The ultrastructural characteristics of *R. venosa* egg capsules were analysed starting from observations of their antifouling activity and mechanical resistance to currents in mid-shore areas. To the best of our knowledge, this is the first study on *R. venosa* capsules with the aim of better understanding their structural and compositional adaptations. This information can shed light on the reproductive biology and remarkable hardiness of this invasive species. The novelty and importance of this study also arises from elucidating the ultrastructural composition of the capsules, which impacts the taxonomy and the evolution of mollusc species or of biomaterials models.

## Results

### Egg capsule surface observations, morphological characteristics and histochemical analysis

**Figure 1:**
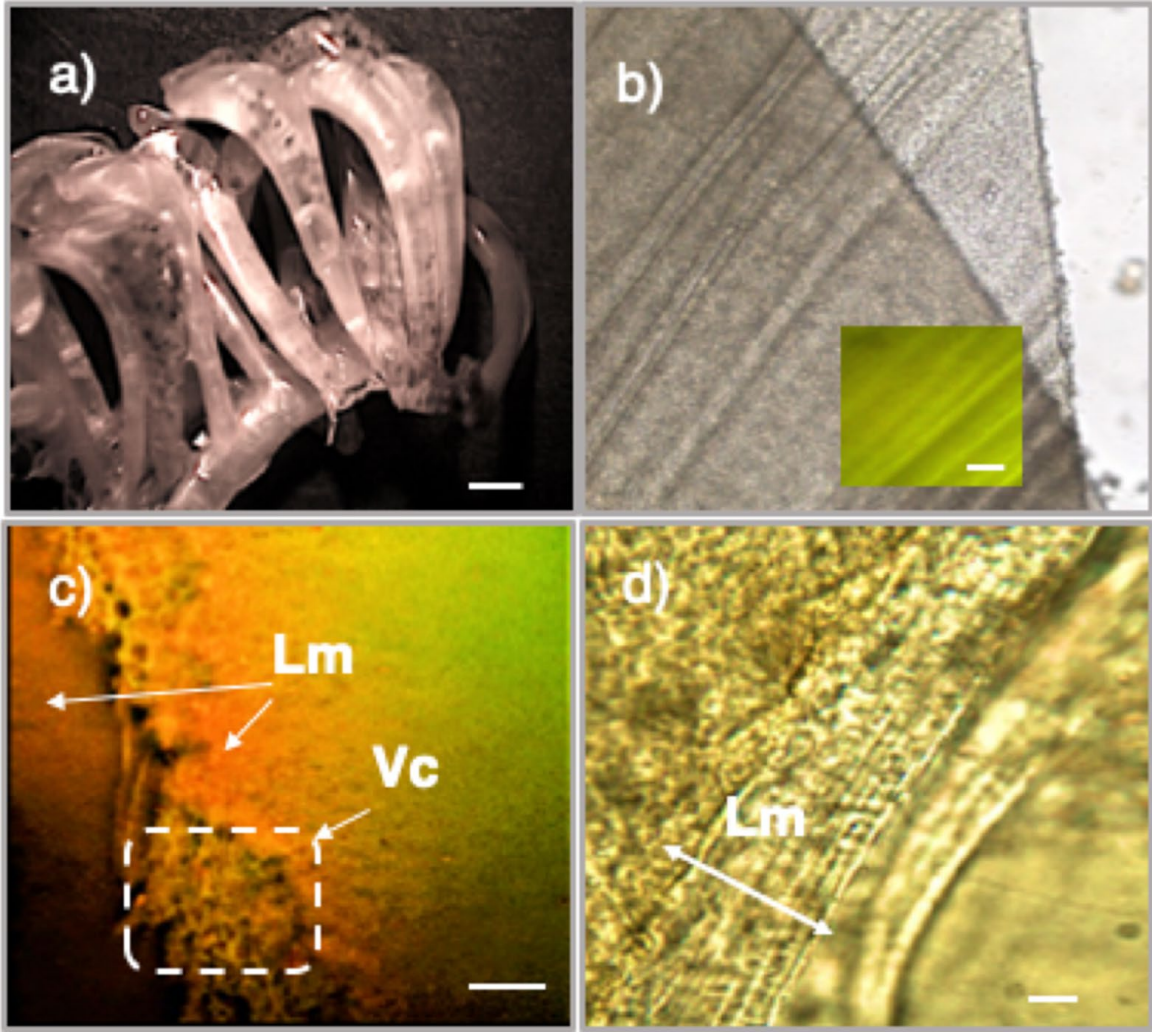
The morphology of the capsule of *R. venosa* eggs. (**a**) General appearance of the egg capsules; scale bar = 2 mm; (**b**) The capsule surface features; the small image shows the observed autofluorescence; scale bar = 10 μm; (**c**) The edge of the transverse section, Vc = vacuole mesh, stained with acridine orange; scale bar = 5 μm. (**d**) Lamellar layer (Lm) array in the section line; scale bar = 1 μm.

Egg capsules of *R. venosa* have a leathery wall texture. They are flexible, with a high degree of elasticity, and maintain their shape even after drying. The capsule body is 10–18 mm long and has apical aperture for planktonic larvae hatching (Fig. 1a). The outside of the capsule surface shows repeat folds (Fig. 1b).

The capsule walls exhibit complex structures (Fig. 1c,d) with regular, lamellar and multi-layered surfaces (Fig. 1d—Lm). A vacuolar mesh (Fig. 1c—Vc) was identified as the median layer. The vacuoles have different shapes and sizes (Fig. 1c).

Autofluorescence was observed when microscopic studies at an excitation wavelength of 450–480 nm with an emission wavelength of 515–520 nm (green filter) were carried out to evaluate the structure (Fig. 1b).

**Figure 2:**
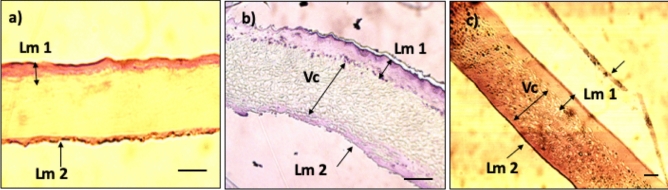
*Rapana venosa* egg capsule transverse section areas and structural characteristics analysed using various staining procedures; (**a**) PAS staining; scale bar = 10 µm. (**b**) Acetic carmine staining, details of lamellar layers (Lm), vacuoles (Vc); scale bar = 10 µm; (**c**) H&E staining; scale bar = 10 µm (Lm 1 = superficial external layer; Lm 2 = internal layer).

Histochemical analyses provided evidence of the differences between the superficial layers (Lm) and the median, which contained the vacuolar mesh (Vc). This type of measurement was performed by staining techniques (PAS, acetic carmine and H&E). The thickness of the wall was between 40 µm ± 0.35 µm and 52 ± 0.5 µm and depended on the size of the capsules. The outer layer (Lm 1) appeared as a well-structured layer with a more consistent architecture thickness (10 ± 0.25 µm) than the inner layer (Lm 2). The internal layer was folded and thin, with a thickness between 2 and 4 µm. This last layer appeared more when stained with PAS and H&E (Fig. 2a).

When PAS was used for staining, a superficial layer in the wall composition was observed (Fig. 2a, Lm 1 and Lm 2). The H&E (haematoxylin and eosin) stains allowed us to observe the egg capsule structures as dark red shades (Fig. 2c), revealing a non-discriminating distribution of the proteins. Eosin, which stains proteins nonspecifically, was pink^13^.

The observed exfoliated superficial material on the outer side of the capsule surface (Fig. 2c) can be explained by the lamellar structure of the superficial layer.

By combining these staining techniques, the thickness of the layers and the changes in the ratio between the Vc layer and Lm layers were easier to observe and distinguish (Fig. 2b,c).

**Figure 3:**
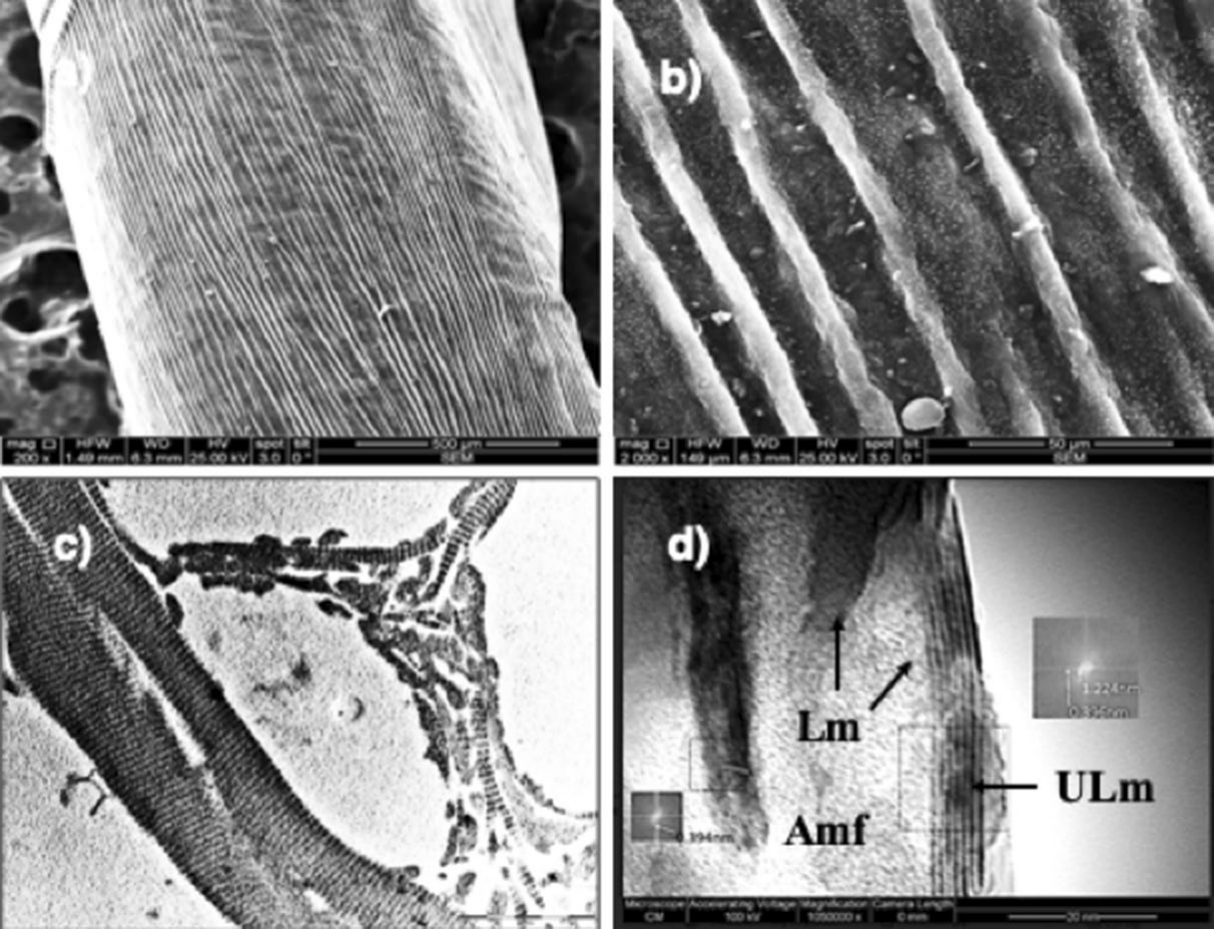
Structural and ultrastructural features of *R. venosa* egg capsules; (**a**) SEM image of the capsule; scale bar = 500 µm; (**b**) Surface capsule details, SEM image; scale bar = 50 µm; (**c**) Details of the capsule wall ultrastructure (cross-section TEM image), a lamellar system with striations (arrow); scale bar = 500 nm; (**d**) Ultrastructural details with parallel oblique lamellar orientation (Lm) and ultra-lamellar layers (ULm); scale bar = 20 nm.

The folds shown in the egg capsule were also investigated at higher magnification by SEM (Fig. 3a). The distance between the folds was between 15 and 30 μm, while the thickness of these folds was between 10 and 12 μm (Fig. 3b).

By analysing the TEM cross-section, a striated structure of the ultrastructural layer was observed (Fig. 3c). The electron microscope images (Fig. 3c,d) showed that the lamellar bands (Lm) had a microfibril ultrastructure (ULm) with oblique dispositions. These layers (Lm) existed in an amorphous system (Amf). The electron microscope images (Fig. 3c) allowed for the visualization of the arrangement of the filamentary material, which revealed the presence of striated strips. Similar images were described in the arrangement of chitin microfibres from the procuticle of arthropods^14,15^.

Details are shown in a high-resolution image (Fig. 3d) in which the size of the fringe width was 1.22 nm. The image shows that the microfibrils are arranged in a highly organized parallel crystalline structure, the reason for which is still unclear.

### Mechanical, viscoelastic and swelling properties

**Figure 4:**
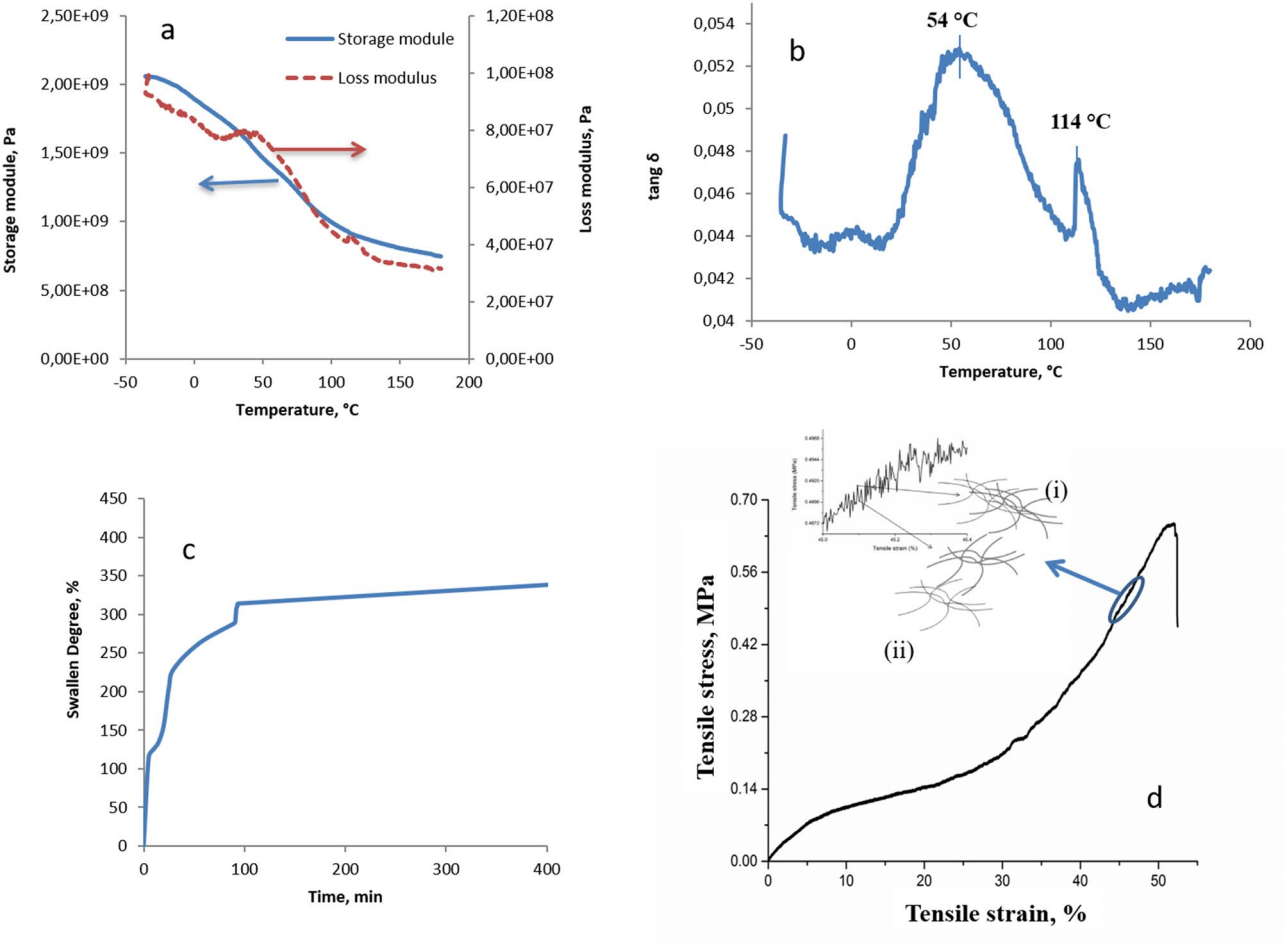
Mechanical, viscoelastic and swelling properties of *R. venosa* egg capsules: (**a**) storage and loss; (**b**) tang δ as a function of temperature; (**c**) swelling degree versus immersion time; (**d**) tensile stress behaviour.

As seen from the evolution of the three parameters (Fig. 4a,b), the material was a blend between two polymer compounds. This statement is supported by the presence of two peaks in the loss angle tangent (tang δ) versus temperature plot and the loss modulus versus temperature plot; the difference between the temperatures corresponding to the peaks was not very large: 54 °C (tang δ) compared to 45 °C (loss modulus) and 114 °C (tang δ) compared to 112 (loss modulus). The presence of these two maxima revealed an immiscibility of the two major compounds of the material (chitosan and proteins), although the elastic response indicated a constitutive coherence. As a material of biological origin, the constituent sequences of the macromolecular nature were accompanied by a series of small molecular compounds acting as plasticizer/crosslinking agents, which can determine the zonal organization of the superstructures in crystalline type formations.

The response of the material to a tensile stress at a constant rate (0.1 mm/min) showed the typical behaviour of plastic materials with high flexibility, suggesting reversible deformations slightly greater than 50% (Fig. 4d). The ultimate tensile stress, which corresponds to the destruction of macroscopic and microscopic integrity, had a value of 0.7 MPa. The value of the Young modulus estimated in the initial linear area (0.25–0.05% elongation) was 0.025 MPa, indicative of a high stability of the biomaterial.

An extremely important aspect, noticeable in the sequential analysis of the mechanical curve, is that the tensile fluctuations occurred with maxima and minima. The only explanation for this observation is a molecular reorganization at the superstructure level, which is expected in biological materials.

The interpretation of this phenomenon (highlighted, in detail, in Fig. 4d) consisted of a cascade of local “micro-breaks” occurring under elongation, which were not attributed to the destruction of chemical structural bonds but to the destruction of bonds at the supramolecular level – in particular physical bonds. Any “rupture” induced the formation of other interactions in the immediate vicinity supported by the chemical nature of the surrounding environment in this material with protein consistency.

This cascade of local “breaks” and reorganizations allows the material to take over the mechanical effort, using it as a source of regrouping and providing flexibility to the ensemble. The apparent material elasticity was given by an elongation up to 50%.

Initially, this fluctuation was attributed to the capacity and sensitivity of the load cell used to capture the tensile load, but when these results were correlated with the DMA results, where a phase separation was evident, it was concluded that a cascade-type mechanism occurred in this case, in which the local “micro-breaks” occurring under elongation were not the result of chemical bond destruction but resulted from a destruction of bonds at the supramolecular level—in particular physical bonds—that determine the crosslinking between the protein and glucosides phases. This conclusion is also supported by the fact that the tested material is a natural material and not two phases (separately).

Thus, in our opinion, the micro-oscillations that occurred at low traction speeds were due to the reorganization of the immiscible polymeric elements that ensured the coherence of the material: the material could withstand a stress load to the maximum energy point corresponding to the initial organization form (Fig. 4d, (i) detail). Once this maximum was reached, a structural transformation with a higher energy state occurred, which was macroscopically unfavourable with respect to the original architecture. As a result, due to the creep specific to the immiscible blends, the constituent polymeric sequences underwent a reorganization, which could be interpreted by conformational chain translations. Thus, the assembly, using the energy accumulated at the maximum point, acquired a new energy level that provided stability (Fig. 4d, (ii) detail). From a structural point of view, a new minimum was reached, ensuring the thermodynamic stability of the assembly. This thermodynamic stability was observed in the structural level by the minimum value of tensile stress. This phenomenon propagated throughout the test range with corresponding elongation shifts until an energy threshold was reached, which destroyed the intermolecular structuring bonds.

The correlation between the tensile stress and the DMA results was attributed to the contribution of the rotational movements of the absorbed water molecules to the rheological changes reported in the material and was observed when a swollen sample was tested. In summary, this material showed flexibility in its dry state, and the flexibility increased with increasing degree of hydration.

### Elements in the capsular surface by EDX mapping

**Figure 5:**
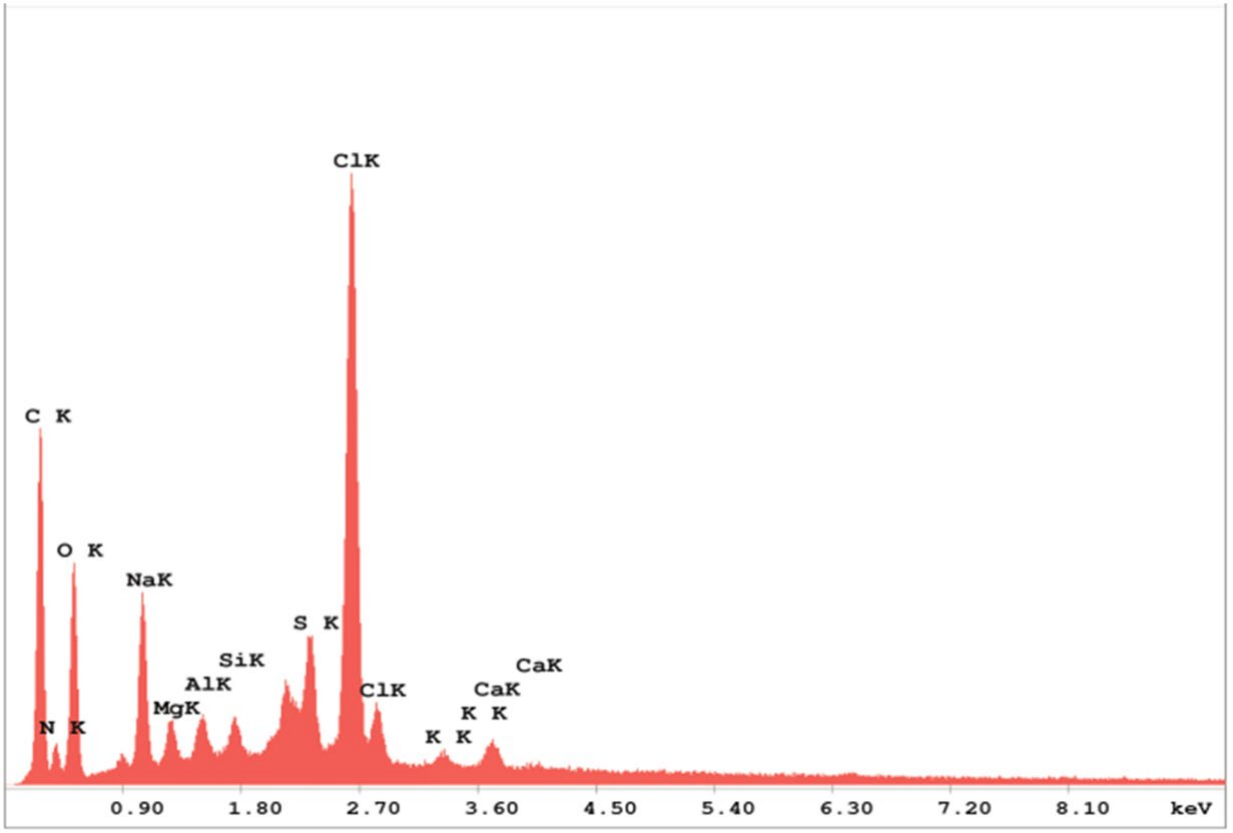
Element distribution in the capsular surface of *Rapana venosa* using SEM–EDX microanalysis.

The quantitative distribution of carbon, nitrogen, oxygen, sodium, magnesium, silicon, chloride, sulfur and calcium elements was evaluated on the surface of the studied capsules using SEM with energy dispersive X-ray (EDX) microanalysis (Fig. 5).

The highest average values were identified for carbon with 51.12 wt%, nitrogen (11.01%) and oxygen with 22.83 wt% (as main components), as shown in Table 1.

**Table 1:** Distribution of the elements, expressed as weight (Wt) and atomic (At) percentages, identified on the capsular surface of *Rapana venosa* by SEM–EDX microanalysis.

Element	wt (%)	At (%)	K-ratio
C	51.12	60.83	0.1333
N	11.01	11.23	0.0112
O	22.83	20.40	0.0290
Na	5.12	3.18	0.0125
Mg	1.01	0.59	0.0031
Al	0.72	0.38	0.0029
Si	0.50	0.26	0.0028
S	1.29	0.58	0.0105
Cl	5.96	2.40	0.0499
K	0.12	0.04	0.0010
Ca	0.31	0.11	0.0028

### Egg capsule composition and characterization

In the structure of *R. venosa* egg capsules, the following components were identified: soluble proteins (46.66 ± 1.00%), chitosan (36.94%) and other components probably consisting of insoluble compounds (15.20 ± 0.80%).

The existence of chitosan in the solid sample (chitosan assay I) was confirmed; as a result, this polymer was found in the structure of the capsules with an average quantity of 11.65 ± 0.60% in the superficial layers (Table 2). The analysis of solutions obtained after deproteinization (chitosan assay II) revealed a chitosan content of 25.29 ± 0.40%.

**Table 2:** Quantitative composition of chitosan from *R. venosa* egg capsules.

Components analysed	Percentual composition* (%) ± SD
Protein (Lowry assay)	46.66 ± 1.00
Chitosan assay I—capsule chemically untreated	11.65 ± 0.60
Chitosan assay II—after deproteinization	25.29 ± 0.40
Ash	15.20 ± 0.80

*Medium values, in dry substance (D. S); D.S was obtained after capsule desiccation for 3 h at a temperature of 65 °C.

Our previous structural analysis^16–18^ using infrared spectroscopy (FT-IR) in transmission mode performed on *R; venosa* egg capsules and on chitosan samples obtained from *R. venosa* egg capsules after deproteinization (R-Ch) and from shrimp waste (P-Ch) and on commercial chitosan (C-Ch) showed the presence of stretching vibrations assigned to carbonyl from the acetamide group of chitin in the spectra of the C-Ch and P-Ch samples. Compared with those spectra, small shifts and weaker bands were observed in the spectra of R-Ch and *R. venosa* egg capsule samples, in which the vibrations were assigned to the carbonyl groups of amides. Additionally, stretching vibrations specific to amino groups in the structure of chitosan were identified in the spectra of all samples, while in the spectra of R-Ch and *R. venosa* egg capsules, the absorption bands were less intense^17^. The peaks between 1613 and 1683 cm^−1^ (Table 3), detected in the spectra of all chitosan samples, were assigned to the amino groups specific to the chitosan structure^19,20^.

**Table 3:** Characteristics of capsules and chitosan powder extracted from gastropod capsules (R-Ch).

	Characteristic analysed			
	Solubility	Fluorescence* (emissions colour)		FT-IR Spectral assignment (cm^−1^)
Capsules	Alkaline solution pH > 9	YG	R	1698
Extracted chitosan (1.5–3 kDa)	Acid solution pH 4–6	YG	R	1683 1613

*Epifluorescence microscopy analyses, blue filter (λ_ex_ = 450—490 nm; λ_em_ = 515–520 nm); green filter (λ_ex_ = 510–550 nm; λ_em_ = 590 nm); YG = yellow-green, R = red.

The chitosan obtained from *R. venosa* egg capsules had a molar mass of 1.5–3 kDa, which was significantly lower than the molar mass of chitosan obtained from shrimp (350–490 kDa) or the molar mass of commercial chitosan (50–190 kDa)^18^. In addition, its solubility in acetic acid (1%) and its toxicity were different than those of standard chitosan^17,18^.The capsule structure showed fluorescent emissions (Table 3) of yellow-green (λ_ex_ = 450–490 nm, λ_em_ = 515–520 nm) and red (λ_ex_ = 510–550 nm; λ_em_ = 580–590 nm). The same effect was observed for granular chitosan obtained from these capsules (after deproteinization).

## Discussion

The study of the capsule structure revealed regular folding of the external surface, which was visible in both optical and electron microscopy. The capsule size, wall thickness, wall structure, and surface morphology could affect the resistance of the capsule wall and the boundary layer to water loss^8^. In *Rapana venosa,* the wall thickness of 40 µm ± 0.35 µm and 52 ± 0.5 µm (Fig. 2) was correlated with the capsule resistance to water loss. Similar observations of the intertidal gastropod *Nucella emarginata* showed that the wall thickness of the egg capsules varied from 53 to 79 µm, and the egg capsules exhibited resistance to water loss^8^ from 22.4 to 33.6 s/m. The tides are associated with the withdrawal of sea water, which would favour the exposure of gastropod larvae to UV radiation and desiccation. Some intertidal species deposit egg clusters in habitats that are unprotected from sunlight^21^; therefore, UV radiation may inhibit the microfouling of egg masses of this species that spawn in exposed intertidal habitats^22^.

The autofluorescence of the wall of the *R. venosa* egg capsule (Fig. 1b) was correlated with the elements that constituted the capsule composition, which, in turn, were associated with microclimatic protection of the capsules for larval adaptation during development periods. The role of autofluorescence was studied and correlated with UV protection^8^ in the eggs of molluscs. Autofluorescence provides UV protection and a form of communication for organisms living in different environments, such as shrimps^23^, insects^24^ and spiders^25^.

The wrinkled capsule surface (Fig. 1a) and the ultra-folding arrangement identified at high magnification (> 2000) (Fig. 3b) had a considerable significance in protecting larvae. This arrangement could be responsible for preventing microorganism, including micro- and nanoplankton, and inorganic particle adsorption onto the surface. Many irregular folds that allow antifouling protection during the first few days of embryo development have also been observed on the surface of *Sepioteuthis australis* egg capsules (Cephalopoda, Loliginidae)^26^. Capsular surface microtopography with inter-waviness distances between 15 and 20 μm (Fig. 3b) limited the possibility of adhesion and generated contact points with macro- and microfouling-sized organisms. Surface roughness and waviness profiles were positively correlated with increased fouling resistance^27^. Studies on the microtopography of wrinkled surfaces, ranging in size from a few nanometres to millimetres, have highlighted the antifouling effect of wrinkled layers^28^. These wrinkled surfaces are interesting due to their amplitude and density. Surface scratching studies have allowed the development of a concept according to which they are formed in response to critical compressive stress^29^. The size of these wrinkles and the distance between them influence the adhesion properties of the materials^30,31^.

In the transverse section, the walls of the egg capsule overlapped and formed thin superficial layers (Fig. 1c,d), while the median layer was thicker and had a vacuolar appearance (Fig. 2b,c). This configuration of a vacuolar microstructure has been identified in other egg capsules from *Acanthina monodon* (Muricidae)^32^ and *Dicathais orbita* (Neogastropoda, Muricidae)^9^ as well as in capsules of gastropods from deep-sea hydrothermal vent environments^33^. The role of these vacuolar structures is not well known, but they could be associated with mechanical protection. The suggested lamellar layer model showed a notable structural convergence to a unique fibril structure that has also been observed in the aragonite organization in shells^34^ or in the collagen organization in bones, as well as in mussel byssus and egg capsule, hair or spun silk^35–38^. In addition, the layout suggests an oblique orientation (Fig. 3d), very similar to the design and formation of calcareous shells^10^. Our studies revealed the presence of crystalline structures (Fig. 3d), whose formation is still not understood. The identified microfibrils (1.22 nm), arranged in parallel and oblique orientations (Fig. 3d), suggested a supporting role of the surrounding amorphous material. These micromorphology details could also explain the particular properties of the material, such as the behaviour in different solvents. The 1.22 nm fringes were different from those found in the literature for α-chitin (0.94 nm), β-chitin (0.91 nm) or cellulose (0.60 nm)^39^.

A more careful evaluation of the evolution of the three dynamic-mechanical analysis (DMA) parameters (Fig. 4a,b) revealed that the increase in temperature was accompanied by a continuous decrease in the storage modulus, while the loss modulus and the loss factor (tang δ) exhibited three and two peaks, respectively, in the same temperature range. In the tang δ curve, the first peak was located in the range 16–111 °C at approximately 54 °C, and the second peak (111–135 °C) showed a maximum at 114 °C. The first peak represented the phase separation observed in the DMA study. Regarding the loss modulus, peaks occurred at 35.5 °C, 45 °C and 112 °C. The first peak in the tang δ curve was broad and centred at 54 °C. This peak represented the peak observed in the loss curve at 45 °C which was shifted, and because it was a broad peak, it overlapped with the peak observed at 35.5 °C in the loss modulus curve.

The third peak obtained in the loss modulus curve was observed at 112 °C (similar to that obtained in the tang δ curve). Correlating the values obtained with similar experimental data^40^, the peak at 112 °C can be attributed to the α-transition corresponding to chitosan Tg. The chain mobility of chitosan also increased because of a decrease in the intermolecular interactions due to dissociation of hydrogen bonds, which started the process of molecular chain scission due to the breaking of glycosidic bonds.

In the literature^41–43^, the transition at low temperatures is generally associated with local motions of side groups in chitosan, and by comparison with the broad relaxation of pure polysaccharides highlighted by dielectric spectroscopy at low temperature (− 120 to − 5 °C) as the result of the orientational motions of local segments via glycosidic bonds, this transition was attributed to β-relaxation. In this context, the peak at 54 °C can be assigned to the protein phase, especially since our sample has a complex composition (demonstrated by other types of analysis) that ensures the coherence of the material. Therefore, the two peaks in the tang δ curve are due to phase separation, a consequence of the cleavage of intermolecular bonds. This phase separation generates a sugar-rich phase and a protein-rich phase^44^. It is believed that the damping peak (observed at 54 °C) is also due to the rotational motion of water molecules^45–48^ and/or the water polymer complex^49^; therefore, the peak position, height and width depend on the moisture content.

The values obtained from the DMA measurements were specific to crosslinked systems. The material tested, the *R. venosa* egg capsule, is a very complex material mainly consisting of physically crosslinked chitosan. Therefore, without water (which helps to plasticize the material), the egg capsule behaviour could resemble that of cellulose, the main component of wood. This fact was supported by the material behaviour in humid environments, which was relevant to the water retention and swelling degree evaluation (Fig. 4c). It should be pointed out that the water absorption saturation was reached within the first 150 min; thus, the material showed a behaviour similar to that of hydrogels with respect to its sensitivity to trace water. The tendency to increase the amount of retained water indicated a potential architectural reorganization process. Because the tested sample was hydrated, we suppose that the formation of hydrogels occurred via covalent bonding between chitosan and the protein chains to form a hydrogel with much higher stability than physically associated hydrogels. However, this approach requires the chemical modification of the primary structure of chitosan, which could alter its initial properties, particularly if amino groups are involved in the reaction. Both the amino and hydroxyl groups of chitosan can form a variety of linkages, such as amide and ester bonding, as well as Schiff base formation^50^.

The elements identified in the capsule membranes of *R. venosa* using energy dispersive X-ray (EDX) microanalysis were carbon, oxygen and nitrogen (as main elements), indicating the presence of proteins and polysaccharides^51^. EDX microanalysis was previously used by Ghannam et al.^52^ to compare samples of chitosan extracted from shrimp and crayfish with commercial chitosan. Peaks related to these main elements and a similar distribution of the elements were reported in the study.

Proteins (46.66 ± 1.00%) and chitosan (36.94 ± 0.50%) were identified in the egg capsule structures. The chitosan proportions in the capsular structure were determined by colorimetric methods. From a total quantity of 36.94%, they were distinguished as follows: the value of 11.65 ± 0.10% corresponded to chitosan on the capsule surface and the value of 25.29 ± 0.40% was determined after the deproteinization process (Table 2). In addition to the organic elements (C, H, O, N), which are predominant in the capsule composition, the ash content is another important parameter for capsular characterization. By capsule combustion, volatile compounds such as CO_2_, H_2_O, and NO_X_ are released, while the rest of the elements form crude ash. The ash value (15.20 ± 0.80%) obtained from the capsules after the experimental measurements indicated the presence of the major minerals (Na, K, Mg, Ca—observed by EDX measurements) within the lamellar layers. Similar capsular compositions, proteins and carbohydrates, but no lipids, were observed in the *Ilyanassa obsoleta* and *Nucella lapillus* gastropod species^10^. Additionally, 78% proteins and 8% carbohydrates were present in the capsules of *Buccinum*^53^.

The presence of chitosan in *R. venosa* egg capsules has been reported in our previous studies^16,17^. Initially, we believed that chitosan and chitin may be both present in the micromorphology of the walls, with chitosan predominantly found in the superficial layers. The method used to identify chitosan from the solid samples (chitosan assay I) allowed us to confirm the presence of this polymer in chemically untreated capsules. Validation of the presence of a significant amount of chitosan in these capsules by analytical methods led to the conclusion that this structure may be correlated to the properties of this biopolymer. Certain studies indicated the presence of different types of components in the wall capsule^10^: proteins, carbohydrates, mucopolysaccharides, glycoproteins, and plastic polymers^36^. Structures with similar features have been observed in the biopolymers identified in the *Busycon canaliculatum* and *Kelletia kelletii* species of Caenogastropoda^37^. We believe that the formation of this type of chitosan is possible at the level of gonoduct structures at the time of capsule deposition in the marine environment. Moreover, our hypothesis supports the possible transformation of chitin into chitosan through chitin deacetylase enzyme activity at the gonoduct level to provide specific flexibility for capsule formation.

In the living world, the formation of chitosan, which possesses superior properties compared to chitin, is a survival strategy, although the chitin-to-chitosan transformation results in a loss of mechanical strength of the structure and sensitization to certain environmental factors^53^. Chitin deacetylases are secreted during an exclusive period corresponding to their special biological roles. This enzyme and chitosan have been studied in both eukaryotic fungi and insects^54–56^. For example, in fungi, the transformation occurs either during vegetative growth^57^ with a role in cell wall synthesis (*Mucor rouxii, Saccharomyces cerevisiae*) or during the penetration of the plant wall by the hyphae of the fungus (*Colletotrichum lindemuthianum*). In fungi, chitosan increases the spore resistance^57^ against various environmental factors, such as acidity, alkalinity, temperature-induced stress, or salts in the environment^58^, compared to vegetative cells. Additionally, chitosan enables the adaptation of spore survival in the digestive tract of insects, as indicated by studies on *Saccharomyces cerevisiae* and *S. pombe*, which maintain viable spores after ingestion by *Drosophila melanogaster*^59^*.*

The presence of chitin was identified and characterized in various species and supported many organisms during the Cambrian life explosion^60^. In recent studies, chitin was identified in fossilized gastropod egg capsules dating back over 200 million years to the beginning of the Jurassic period^61^. Moreover, chitin was found in prosobranch molluscs in epithelial cells (where it was identified intracellularly), playing a role in defence^62^ or in the prismatic layers of mollusc shells. Other studies showed the role of chitin in symbiosis with *Vibrio fischeri* by its presence in the blood cells of squid haemocytes, *Euprymna scolopes*^63^.

The structure of the *R. venosa* egg capsules that were analysed in this study at the microstructural level showed complexity in terms of the filament layout and the microfibrillar composition; furthermore, the presence of chitinous structures was confirmed. The specific properties (mechanical, chemical, antifouling and optical) could explain the reproductive efficiency and the spread of the species with remarkable adaptations of this organism.

## Methods

### Sample preparation and microscopy

*R. venosa* egg capsules were collected by scuba diving at a depth between 2 and 25 m along the Romanian Black Sea shore. The capsules were collected without viable embryos representing an epibiotic waste material. Microscopic observations were performed directly on the capsular fragments (on wet and dry samples) to observe the capsular surface. The cleaned capsules were then washed and placed in fixation solution (ethyl alcohol or glutaraldehyde). After paraffin coating (histochemistry) and inclusion in epoxy resin (electron microscopy), ultramicrotome sections were obtained. To label specific structures, several staining techniques were used: acetic carmine, haematoxylin–eosin (H&E)^13^, the periodic acid Schiff technique (PAS), and acridine orange stain. For electron microscopy observations, capsule fragments were processed as follows: prefixation in 1 M cacodylate buffer solution with 2.7% glutaraldehyde (GA), followed by fixation in 2% osmic acid solution (in small glass tubes containing 2 mL of cold fixation medium, consisting of 2% (w/v) osmic acid (OsO_4_) dissolved in twice-distilled water). The fixation was carried out for 1 h at a temperature of 4 °C. For desiccation, serial baths of 30%, 50% and 70% cold ethanolic solutions were used. The samples were maintained in each of the ethanolic baths for 10 min. For inclusion in epoxy resins, the samples were placed in a mixture of Epon 812 (consisting of DDSA, MNA and DMP30 as polymerizing agent) and propylene oxide at an Epon 812:propylene oxide ratio of 1:1 (v/v). The fine sections were double-stained with uranyl acetate and lead citrate and lead acetate solutions and then examined by SEM and TEM. A scanning electron microscope (SEM FEI QUANTA 250 equipped with an energy dispersive spectrometer) and a transmission electron microscope (TEM-Microscope Tecnai T12) were used. Optical microscopy observations were performed with an epifluorescence microscope (OPTIKA B – 350); blue filter (λ_ex_ = 450–490 nm; λ_em_ = 515–520 nm); green filter (λ_ex_ = 510–550 nm; λ_em_ = 590 nm). The captured images were processed with Optikam Pro 3 Software.

### Mechanical and viscoelastic properties

Mechanical properties were determined with an Instron 3382 Universal Testing Machine with video extensometer, and the result was the average of five tests for each specimen. The tests were carried out at room temperature with a crosshead speed of 50 mm/min, and the samples were previously hydrated in demineralized water for 24 h. The size of the samples was 10/5/2.5 mm. All measurements were performed at room temperature, and the tensile strength (TS) and ductility (percent elongation EL%) of the films were calculated using the following equations:

$$TS = \frac{Load\;at\; break}{{Original\; width \, \times \,original\; thickness}}$$

$$EL \% = \frac{Elongation\; at \;rupture}{{Initial \;gage\; length}} \times 100$$

Dynamic-mechanical analysis (DMA) tests were carried out with TRITEC 2000 B equipment (Triton Technology, UK). The dried samples were analysed in single cantilever bending mode at 1 Hz frequency. The data were collected from room temperature (RT) to 180 °C at a heating rate of 5 °C/min.

The dry samples after washing had the following dimensions: length, 10 mm; width, 5 mm; thickness, 2.5 mm. The obtained samples were washed to remove solid impurities (traces of sands). No surface-finishing technique was employed. Fractions of different egg capsules were mixed, and the measurements were performed on samples that were randomly chosen. These samples were further handled in dry nitrogen as much as possible to reduce the chance of moisture reducing the glass transition temperature. Samples were measured in triplicate, and the values were averaged. Tensile strength, elongation, and Young’s modulus are parameters that relate the mechanical properties of the samples to their chemical structures. The tensile strength expresses the maximum stress in a sample during tensile testing.

Generally, dynamic-mechanical thermal analysis (DMA) of polymeric materials is a very important technique due to its high sensitivity in detecting changes in the internal molecular mobility and in probing the phase structure and morphology of polymers. The dynamic mechanical properties, such as storage modulus (E′), loss modulus (E), and loss tangent (tang δ), of polymer blends are sensitive not only to different molecular motions but also to various transitions, relaxation processes, structural heterogeneity, extent of crosslinking and the morphology of multiphase systems.

The characteristic of DMA spectra is that the loss modulus E or loss factor tang δ = E′/E attributed to the relaxation processes α and β, respectively, can show several peaks. If the β-process is always assigned to the local mode of relaxation in the amorphous phase, the α-relaxation is strictly related to the glass transition of the amorphous phase, which is determined by both intra- and intermolecular interactions. Consequently, when the temperature increases, this relaxation is accompanied with a continuous decrease in the storage modules E' and the presence of a peak in the E curve. The investigation of molecular behaviours over a broad temperature range can be used to evaluate the molecular response of a mixture (homogeneous/heterogeneous) of macromolecules with different chemical natures – polymer blends. In the case of heterogeneous (phase-separated) mixtures, the molecular motions of the components remain unchanged, while in homogeneous mixtures, the motions should be strongly affected and show a single α-transition. The data obtained over a broad temperature range can also be used to evaluate the molecular response of a polymer in blends with other polymers^64^.

The swelling experiments consisted of immersing one dried sample of approximately 9.6 mg into 500 mg of water and measuring the mass of the sample at specific time points. The experiment was performed in triplicate, and the average value was used. The swelling degree was calculated according to the equation:

$$SD(\% ) = \frac{{\left( {W_{s} - W_{d} } \right)}}{{W_{d} }} \times 100$$

where W_d_ is the mass of the dried sample and Ws is the mass of the swollen sample.

### SEM- Energy dispersive X-ray (EDX) microanalysis

The elemental analysis of the capsular surface was performed by the SEM–EDX (ADC1 detector at 182 points, 80,000 magnitude, 30 keV, Dwell time 200 ms).

### Chemical and materials for protein quantitation

Sodium hydroxide pellets (Chimreactiv, Romania), copper (II) sulfate-anhydrous powder (Merck KGaA, Germany), sodium carbonate-anhydrous powder (Merck KGaA, Germany), sodium potassium tartrate tetrahydrate powder (Spectrum Chemical MFG CORP), Folin-Ciocalteu phenol reagent (Merck KGaA, Germany) and bovine serum albumin-lyophilized powder (Sigma Aldrich, Germany) were used during the experiments.

### Protein assays

The data analysis and processing were performed according to a modified Lowry protocol^65^. The capsules were milled and 0.6183 g were added to alkaline extractive solutions 1:40 (w/v) for 4 h. After centrifugation (10.000 rpm), the alkaline supernatant was recovered and subjected to quantitation analyses of the soluble proteins according to the Lowry procedure. Sample dilutions of 1:10 (SD1) and 1:100 (SD2) (v/v) of the alkaline supernatant and distilled water were prepared. Volumes of 0.1 mL from these dilutions SD1 and SD2 were taken and mixed in Eppendorf tubes with the amounts of reagents according to the Lowry procedure. For the calibration curve, a stock solution of albumin (2 mg/mL, bovine serum albumin BSA) was used. The samples were measured at 750 nm using a Jasco V-630 spectrophotometer. The measurements were performed in triplicate.

### Ash content

The ash content (Ash) of the egg capsules was determined using MICROTERM 1206, a laboratory muffle furnace, according to ASTM F 2103. To determine the ash content from the egg capsule samples, a small quantity of cleaned, washed, dried and fine-cut egg capsules was placed into previously ignited, cooled and tarred porcelain crucibles. The ash content (%) of samples was determined at 800 °C in triplicate using the following equation:

$$\% Ash = \frac{{Mass\;of\;residue \left( {\text{g}} \right)}}{{Mass\; of\; initial\; sample \left( {\text{g}} \right)}} \times 100$$

### Chitosan quantification by colorimetric assay

A chitosan colorimetric assay kit (K995-100) BioVision, USA, was used. This is the first commercial kit available for measuring chitosan content from both solid and liquid samples. The capsules were washed with distilled water, dried and weighed (50 mg), and introduced into chitosan analysis solutions following the analysis procedure for solid samples (chitosan assay I—solid sample) according to the determination kit. Additionally, the presence of chitosan was tested after deproteinization (chitosan assay II—liquid sample) using the protocol for liquid samples (K995-100) in extraction solutions.

### Spectral measurements -FTIR chitosan identification

The chitosan identification was performed using infrared equipment from the Interspectrum, Interspec 200-X model in the wavenumber region of 4000–400 cm^-1^ at a resolution of 4 cm^-1^. Spectral analyses were carried out with the chitosan extracted from the capsules^66^. For comparison by spectral investigation and physical–chemical characterization, two chitosan samples were chosen as reference: commercial chitosan (C-Ch) with a molar mass *M*_*w*_ = 50–190 kDa (based on viscosity, *t* = 25 °C) with product number 448869, CAS: 9012-76-4 purchased from Sigma Aldrich and chitosan extracted from shrimp waste (P–Ch) with a molar mass *M*_*w*_ = 350–490 kDa (based on viscosity, *t* = 25 °C). The method used to determine the molar mass was based on intrinsic viscosity determination of the chitosan dissolved in dilute acidic solutions^67^. An Ostwald-type capillary viscometer was used for these measurements.

### Physical–chemical and optical characteristics of the extracted chitosan powder

The solubility of the chitosan samples was tested in acetic acid solutions (1%) at *t* = 25 °C in triplicate by applying a modified and improved method of Fernandez-Kim^68^. As equipment, a VELP—Digital IR Vortex mixer and a centrifuge were used. Epifluorescence microscopy analyses were used for particle observation.

### Ethical statement

The species *Rapana venosa* does not have a legally established protection status at the national or European level and therefore is not subject to the procedure for approving the derogation from the protection measures for scientific purposes. Additionally, considering that very small quantities of non-living parts, which can be classified as animal remains without value, were harvested, this study did not require any authorization procedure by the competent bodies.
